# Delivering primary eye care in the 21st century

**Published:** 2022-03-01

**Authors:** GVS Murthy

**Affiliations:** 1 Director: Indian Institute of Public Health, Hyderabad, India.

**The changing epidemiology of eye conditions calls for reimagining primary eye health care services with strong referral linkages.**

**Figure:**
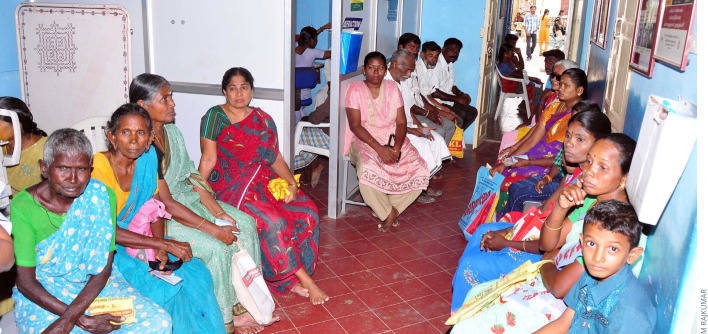
Patients waiting in a community eye clinic. **THIRUMANGALAM, MADURAI** M RAJKUMAR

The World Health Organization (WHO) made a clear, strong call for equity in health care in the ambitious Alma-Ata Declaration (1978), which recognised primary health care as an essential means to achieve health for all by 2000.^1^ Forty years on, despite the changes in the epidemiology of health conditions, the 2018 Astana Declaration reiterated that universally accessible, safe, quality, and affordable primary health care is an integral part of a health system.^2^^,^^3^ Primary health care was upheld as the foundation for universal health coverage and the Sustainable Development Goals. The use of technology in primary health care was also felt to be important in the current and future contexts.

## Primary eye health care and changing needs

The WHO describes the basic clinical care provided in frontline health facilities as ‘primary care’. Applying this to eye care, the clinical eye health services in primary level facilities should be called ‘primary eye care’ (PEC). We should broaden the scope to achieve ‘primary eye health care’ (PEHC), which includes health promotion, disease prevention, and rehabilitation of the visually impaired. The health systems framework is a helpful template, which can be adapted for assessing primary eye care needs and delivery, as depicted in Table 1.^4^ For example, a district in India has an average population of 1.25 million. For every 100,000 population, there is a health facility called a community health centre, where a paramedical ophthalmic officer/ophthalmic assistant is posted. The person provides refraction services, follow-up services for those who have undergone cataract operation, and clinical support to diagnose eye problems such as a red eye. This function is purely an individual patient-related service at a frontline health facility and can be aptly called ‘primary care’. Such individual patient-related services are also provided by other trained personnel in the private sector. However, the paramedical ophthalmic officer or assistant also provides school vision screening services, engages in health education and communication with trained general health personnel working in the public health system, and provides other eye care activities at the population level. Such services, which are more comprehensive than pure clinical work, constitute primary eye health care activities.

**Table 1:** Primary eye health care in the health systems framework

**Health systems building blocks**	**Possible primary eye care activities**
**Service delivery**	Effective, safe, and quality services, which respond to the needs of communities Affirmative action to ensure access to disadvantaged groups Eye health promotion, preventive care, and rehabilitation
**Health workforce**	Adequately trained community-based salaried eye care workers Volunteer core group supported by facility-based secondary level eye care personnel, including ophthalmic nurses, optometrists, and ophthalmologists Community-based organisations to support PEC
**Health information management systems**	Systems for collecting eye-related data Monthly collection of disaggregated data on all ocular conditions, analysed locally and transmitted to a higher level for compilation Regular feedback provided to primary eye health staff Periodic surveys to assess the magnitude of eye conditions, discern trends, and capture community perceptions
**Medical products and technology**	Clear, simple, and user-friendly manuals on eye care for primary health care workers Equitable access to quality and affordable eye health products in primary care facilities Use of technology to improve case detection, counselling, knowledge, and follow-up at the primary level Augmenting technology-enabled referral pathways
**Health financing**	Committed budget line for PEHC in health plans Financial support for all eye health procedures, including diagnosis and treatment at secondary and tertiary levels Inclusion of outpatient consultations, spectacles, and assistive appliances in health insurance schemes at all levels of eye health care Inclusion of medicines and procedures needed for primary eye care in health insurance schemes (public and private) Identifying community resources that can support some of the costs of delivering PEHC
**Leadership/governance**	Inclusion of PEHC in national policies/schemes and programmes Allocating at least 50% of the national eye care budget to PEHC Direct walk-in at the secondary level only for emergencies; otherwise, consultation through a referral from PEC services, wherever possible, to ensure adequate care is provided Review of existing health programmes to assess the scope for inclusion of eye care services in existing health care programmes, like the non-communicable diseases programme, child health programmes, school health programmes, care of the elderly programmes, etc.

## Social determinants of health and eye care

Primary health care addresses the social determinants of health and the direct medical causes of disease causation. Social determinants of health are the factors that influence everyday life and health: education, housing and the environment, income and social protection, working life conditions, and social inclusion. Indeed, it has been estimated that these factors account for between a third and half of health outcomes.^5^ These factors are also relevant to eye care. They can influence whether or not an individual develops an eye condition and is able to access services for treatment; as well as the prognosis and outcomes.

As the major causes of visual impairment can change over time, PEHC needs to evolve to remain relevant to the current eye health needs of the population (as outlined in Table 2). For example, the dramatic increase in diabetes increases the risk of visual loss from diabetic retinopathy (DR), and an increasingly ageing population is at increased risk of glaucoma. Additionally, there is an epidemic of retinopathy of prematurity (ROP) in low- and middle-income countries because of the increased chances of survival of preterm babies. The changing epidemiology of eye conditions calls for reimagining primary eye care service with a robust referral mechanism to link to secondary and tertiary levels of care (Table 3).

**Table 2:** Adapting primary eye health care to current needs

**Elements of primary eye health care**	**Current needs and adaptation**
**Imparting education on eye health problems and their prevention and control**	Effective communication about eye health to encourage health-seeking behaviour is possible through digital technology and smartphones. Families need constant support to deal with emerging conditions like DR and ROP to reduce the risks of visual impairment. Support groups need to be encouraged for DR, glaucoma, ROP, low vision, etc.
**Ensuring proper food and nutrition, especially in low- and middle-income countries**	Low birth weight is an indicator of maternal nutritional status. It is a risk factor for ROP and refractive errors like myopia. Obesity is a risk factor for DR.
**An adequate supply of safe water and basic sanitation**	Trachoma, a leading and preventable cause of blindness, is caused by unhygienic conditions and lack of water. Diarrhoea can lead to vitamin A deficiency in young children, and severe dehydration may lead to cataract.
**Maternal and child healthcare, including family planning**	Increasing survival for preterm babies has increased the risk of ROP, screening for ROP is essential in preterm and low birth weight babies. Counselling should be given on consanguinity, which is a cause for childhood blindness and conditions like retinitis pigmentosa.
**Immunisation against major infectious diseases**	The primary purpose of rubella vaccination is to prevent congenital rubella syndrome. Measles and BCG vaccinations need to be augmented.
**Prevention and control of locally endemic diseases**	Microbial keratitis is common in agricultural communities in low- and middle-income countries. Leprosy is still endemic in India. Iodine deficiency affects colour vision.
**Appropriate treatment of common diseases and injuries, and referral**	It is essential to identify ocular trauma and to take steps for care and referral. People with diabetes and glaucoma should be regularly screened and tracked for treatment compliance. Those with refractive errors, including presbyopia, need to receive prompt and timely treatment.
**Provision of essential drugs**	The list of essential drugs should include specific eye drops and ointments. Availability of drugs like metformin has to be ensured to reduce the risk of vision loss in patients with DR. Antenatal steroid injections should be available at all levels of health care to prevent the risk of ROP.

**Table 3:** Suggestions to tackle emerging eye care challenges

**Eye conditions/ disease-focused services**	**Primary eye care approaches**
**Near vision correction facilities**	Community-level personnel should be skilled to assess vision and prescribe near vision glasses.
**Distance vision correction facilities, including low vision**	Distance vision assessment skills and equipment must be available at the primary level. Referral algorithms for distance vision impairment need to be developed. Follow-up of those advised referrals have to be instituted to improve compliance. The use of low vision devices is required to be monitored.
**Paediatric cataract, strabismus, and congenital anomalies**	Primary care health personnel must be trained to identify poor vision, white pupil, visible congenital anomaly, and an overtly manifest squint in children for immediate referral.
**Diabetic retinopathy**	A community list of persons with diabetes must be maintained. Tools like a DR risk score is critical to identify those at risk of retinopathy, with counselling and referral for a retina examination. Screening for DR using an affordable non-mydriatic fundus imaging system have to be established in clinics managing people with diabetes, with clear directions on the referral pathway, based on fundus findings. There should be an annual follow-up of the individuals screened.
**Glaucoma**	All individuals with poor vision in one or both eyes where the pupil(s) is/are black need to be referred.
**Retinopathy of prematurity**	Screening for ROP should be organised at primary health centres for better compliance. Nurses, female health visitors, and traditional birth attendants need to be trained to counsel mothers of preterm infants about the importance of timely ROP screening in the neonatal unit. Facility-based births should be encouraged, for women about to go into preterm delivery.
**Health communication in primary clinics and the community**	Health communication have to be improved with appropriate communication materials, including digital content. Peer and parent support groups can be used to share experiences and learning.
**School programmes**	School eye health programmes should be implemented for the catchment population.

## The impact of COVID on primary eye care services

COVID-19 has impacted all levels of the health system, particularly primary health care and primary eye care services. Elective services like school vision screening and screening for ROP and DR have been shut down. With the COVID vaccine drive under way in many low-and middle-income countries, all primary health personnel, including eye care personnel, will continue to shoulder COVID-related responsibilities. Also, service users are apprehensive about visiting health facilities. All of this will have a catastrophic cascading effect as those not screened and managed in time may become visually impaired.

In the South Asia region, some services had resumed towards the last quarter of 2020. However, most were suspended during the first quarter of 2021 due to the second surge of COVID from March 2021. Going forward, there is a huge need to reduce risks to both users and providers of primary eye care. There is an urgent need to employ teleconsultation in eye care. Its effectiveness, though, will depend on developing smartphone-based applications to capture images of the back of the eye, which could be shared from the service user's home. Technology, like PEEK,^6^ will need to be used more widely. Each country should look at what works best in its context and plan accordingly.

“Deploying technology can help provide primary eye care and reach the unreached population.”

## References

[1] White F. Primary health care and public health: foundations of universal health systems. Med Princ Pract. 2015;24(2):103-16. 25591411 10.1159/000370197 PMC5588212

[2] World Health Organization. Global conference on primary health care: from Alma-Ata towards universal health coverage and the sustainable development goals, Astana, Kazakhstan, 25 and 26 October 2018. Astana: World Health Organization; 2018. Available from: https://bit.ly/3oSvCJ7 (accessed 10 June 2021).

[3] World Health Organization. Declaration of Alma-Ata international conference on primary health care, Alma-Ata, USSR, 6-12 September 1978. Available from: https://bit.ly/3zBs7M7 (accessed 10 June 2021).

[4] World Health Organization. Everybody's business: strengthening health systems to improve health outcomes, WHOs framework for action. Geneva: World Health Organization; 2007. Available from: https://bit.ly/3gJQEpo (accessed 10 June 2021).

[5] World Health Organization. Social determinants of health. Available from: https://bit.ly/35vPPLL (accessed 10 June 2021).

[6] Bastawrous A. Increasing access to eye care … there's an app for that. Peek: smartphone technology for eye health. International J Epidemiol. 2016;45:1040-3 (accessed 10 June 2021). 10.1093/ije/dyw086 27215615

